# The Gift of Expression

**Published:** 1920-10-09

**Authors:** 


### THE EDITOR'S LETTER-BOX.

**Correspondence on all subjects is invited, but we cannot in any way be responsible for the opinions expressed by our correspondents, who must give their name and address as a guarantee of good faith, but not necessarily for publication. Correspondents are reminded that brevity of style and conciseness of statement greatly facilitate early insertion.**

### "The Gift of Expression."

*To the Editor of* The Hospital.

Sir,—I am grateful to the writer of the "Gift of Expression" for the courtesy of a reply. If expression is a gift, and indeed, as everyone knows, this gift is rare (more especially to women), there are many who have acquired the art to a considerable degree (not necessarily being gifted). I am very glad that the writer agrees with me that we require women with sound judgment at this moment to tackle the problem with which the profession is faced.

Further, I am grateful for the explanation with regard to the rank and file, with which we are all already well acquainted. However, I do not see why a matron or teaching sister—who can hold their nurses by their example and personality, making it easier for them to accept the knowledge about to be imparted—should fail in her standard of ethics, any more than a successful teacher who can hold his pupils to the credit of the individual and the College.

I cannot imagine anything more pathetic than to take this matter to the Press. Just imagine a teacher airing his opinion with regard to apathetic and indifferent pupils. Why, it is enough to make angels weep!

The nurses will accept their responsibilities as they have always done. They have never yet failed us, and they will not fail us now; but it is to the leaders of the profession, the G.N.C. and the College of Nursing, we must look to for reform and to the wise guidance of matrons and superintendents as far as the rank and file is concerned at present.

A College Member.

